# Follow Me! A Tale of Avian Heart Development with Comparisons to Mammal Heart Development

**DOI:** 10.3390/jcdd7010008

**Published:** 2020-03-07

**Authors:** Rusty Lansford, Sandra Rugonyi

**Affiliations:** 1 Department of Radiology, Keck School of Medicine of University of Southern California, Los Angeles, CA 90033, USA; lansford@usc.edu; 2 Department of Radiology and Developmental Neuroscience Program, Saban Research Institute, Children’s Hospital Los Angeles, Los Angeles, CA 90027, USA; 3 Department of Biomedical Engineering, Oregon Health & Science University, Portland, OR 97239, USA

**Keywords:** cardiogenesis, avian embryo, intravital imaging, transgenic quail, optical coherence tomography, time lapse microscopy, laser microscopy, optical microscopy, ultrasound, micro computed tomography

## Abstract

Avian embryos have been used for centuries to study development due to the ease of access. Because the embryos are sheltered inside the eggshell, a small window in the shell is ideal for visualizing the embryos and performing different interventions. The window can then be covered, and the embryo returned to the incubator for the desired amount of time, and observed during further development. Up to about 4 days of chicken development (out of 21 days of incubation), when the egg is opened the embryo is on top of the yolk, and its heart is on top of its body. This allows easy imaging of heart formation and heart development using non-invasive techniques, including regular optical microscopy. After day 4, the embryo starts sinking into the yolk, but still imaging technologies, such as ultrasound, can tomographically image the embryo and its heart in vivo. Importantly, because like the human heart the avian heart develops into a four-chambered heart with valves, heart malformations and pathologies that human babies suffer can be replicated in avian embryos, allowing a unique developmental window into human congenital heart disease. Here, we review avian heart formation and provide comparisons to the mammalian heart.

## 1. Introduction

Birds have been diversifying to inhabit nearly every conceivable habitat on the earth’s surface since the Cretaceous period. With this diversity, birds can flourish in numerous harsh environments, such as flying atop hypoxic Himalayan mountain ranges, swimming deeply in frigid Antarctic waters, and running across hot Mojave Desert sands [1]. To live in such extreme conditions, the bird cardiovascular system (CVS) and respiratory system evolved to systemically deliver sufficient oxygen and metabolic substrates to meet the demands of such severe niches [2,3]. The avian CVS also adapted to efficiently remove metabolic byproducts to maintain cellular function, while maintaining a bird’s body temperature [3,4].

High aerobic activities (e.g., flying) in endotherms (e.g., birds and mammals) require an efficient CVS that is afforded by four-chambered hearts, high systolic pressure and high resting metabolism [3,5]. Both the avian and human heart are located along the midline of the anterior part of the thoracic cavity. The long axis of the heart points slightly to the right of the midline in avians, and to the left of the midline in humans. A positive blood systolic pressure is required to push blood to the body tissues to meet the body’s metabolic needs. Birds have a higher metabolic rate than humans. The average body temperature of a bird is 40–41 °C, while the average body temperature of a human is 37 °C. The resting heart rate of a chicken is about 245 beats/min and can reach ~400 beats/min (the heart rate of the blue-throated hummingbird has been measured at 1260 beats/min), while the resting heart rate of a well-conditioned human is about 60–80 beats/min and may reach ~190–200 beats/min. Birds tend to have larger hearts and pump more blood per unit time than mammals (relative to body size and mass) [6,7,8]. In birds, heart mass (*M*_h_) scales with respect to body mass (*M*_h_) as *M*_h_ = 0.014 *M*_b_^0.91^ [9], whereas in mammals, the relationship is *M*_h_ = 0.0058 *M*_b_^0.98^ [10,11]. This may be due to the high aerobic power input needed to sustain flapping flight. Hummingbirds have the largest hearts relative to body mass of all birds; for 25 species of hummingbirds, *M*_h_ = 0.025 *M*_b_^0.95^ [12], possibly reflecting the high aerobic requirements of hovering flight and that they are so small. Thus, cardiac output is normally greater for birds than for mammals of the same body mass. Such conditions and physiological requirements place inordinate demands on the bird heart, which has to function at a higher level than a human heart.

The avian and mammalian heart transports blood to the lungs and body in a similar manner [13]. Birds and mammals have atrial and ventricular septa, allowing separation between oxygenated and deoxygenated blood, and complete separation of the systemic and pulmonary circulations. The deoxygenated blood returns from the body to the right atrium through the large caval veins. The deoxygenated blood moves to the right ventricle, where it is pressurized for pulmonary circulation. The blood dumps its CO_2_ and acquires O_2_ via the lung capillaries. The newly oxygenated blood returns to the left atrium through four large pulmonary veins, as in mammals. The oxygenated blood moves to the left ventricle, where it is pressurized for systemic circulation. Both avian and mammalian hearts are surrounded by a thin, fibrous pericardial sac that is filled with serous fluid that lubricates the motions resulting from cardiac contractions and also confines the heart so it does not rattle around the thoracic cavity or overfill with blood.

As birds increase their activity level with flight, they must also increase oxygen delivery and thus blood supply to tissues involved with flight. The two main flight muscles, the pectoralis and supracoracoideus, originate in the sternum and insert onto the base of the humerus. Blood to the flight muscles and wings is delivered by the subclavian arteries, which branch into the pectoral (flight muscle) and brachial (wing) arteries. Flight demands lead to an increase in the number of capillary beds in the flight muscles, increasing capillary density. The greater the capillary density, the greater the surface area for gas exchange, at the expense of an increased resistance to blood flow. This requires the heart to pump harder to push the blood through all the blood vessels. Birds that migrate long distances have a greater capillary density (1935 capillaries per mm^2^) in their flight muscles than those of species that do not migrate or migrate only short distances (1604 capillaries per mm^2^) [3,14]. For an outermost example, rufous hummingbirds, which migrate from breeding grounds in Alaska and western Canada to wintering sites in Mexico, have a capillary density of 7000 per mm^2^ in their flight muscles [15].

The ventricles of the bird heart have more muscle mass and less chamber space than those of the human heart. The left ventricle in a bird’s heart is by far the largest heart chamber, powered by a thick cardiac muscle to pressurize the blood for transport throughout the body, and must work especially hard in birds during flapping flight. The right side of the heart only delivers blood to the lungs and the resistance of this circuit is low, thus its smaller size. Externally, the avian ventricles appear trimmer and more pointed than those of the human heart [16]. Internally, the atrial and ventricular walls are smoother than those of the human. The smoother walls and simpler valves of the bird’s heart reduce friction as the blood is pumped through; less friction means less work to pump blood.

In both birds and mammals, up to six aortic arches develop in the embryo, but only three remain in the newborn animal. In birds, the left systemic arch does not develop, and all functions are carried out by the right systemic arch. The blood vessels to the forelimb (subclavian arteries) develop from the anterior arteries supplying the head (brachiocephalic, common carotid, internal carotid arteries). In mammals, the left systemic arch develops, and the right does not. The blood vessels to the forelimbs (subclavian arteries) develop from the dorsal aorta and left systemic arch.

## 2. Avian Models

Studies on avian embryos date back to Aristotle, and even further back, to the Egyptians. Historically, the avian model has played an important role in establishing the foundations in circulation research. Chicken eggs were easy to obtain and could be incubated in ovens [17] to observe different periods of embryo development [18]. William Harvey, for example, used chicken eggs to watch the development of the heart and blood, and was the first to notice the directional flow of blood from the heart into the brain and body during systemic circulation [19]. The existence of capillaries that connected the veins and arteries was confirmed with the aid of a simple microscope by Marcello Malpighi, who also discovered that the heart began to beat before blood started to form [20,21].

Avian models have unique characteristics that make them invaluable for embryonic developmental studies and, in particular, heart development research. First of all, like mammals, chickens and quails are amniotes, animals whose embryos develop within an amnion and chorion, and developmental processes are highly conserved among amniotes [22]. Specifically, the development of the avian heart is similar to that of the human heart [23]. The mature avian heart consists of four chambers with valves as well as inflow and outflow connections (veins and arteries, respectively), and despite some differences, it resembles the human heart [24,25,26,27]. Importantly, cardiac defects found in humans can be recapitulated in avian embryos [26,27,28,29]. Second, like humans, avian embryos remain relatively flat from early to late gastrulation stages [30], enabling time-lapse observation of both dorsal and ventral tissues by means of whole-mount ex ovo culture techniques [31,32]. Pioneering work by María de la Cruz using iron oxide particle labelling in an ex ovo culture, for example, elucidated the details of heart tube formation and the location within the mature heart of primitive cardiac regions from the heart tube [33,34,35]. The whole-mount ex ovo culture technique for avian embryos permits studying the cell behaviors underlying heart and blood vessel morphogenesis under physiological conditions [36,37,38,39]. In addition to labeling with iron oxide particles, heart researchers have labelled cardiac progenitor cells (CPCs) in vivo with radioactive nucleotides [40,41,42,43], vital dyes [44,45,46], and fluorescent proteins [36] to understand their dynamic contributions to heart assembly. The use of transgenic quail embryos that express fluorescent proteins allows following the motion of individual CPCs, as well as extracellular matrix (ECM) components, during cardiac formation. It has been shown, for example, that the myocardium is formed by intercalated CPCs coming from the left and right heart-forming fields [23,47,48]. Third, because avian embryos develop inside the egg, they provide easy access for cell and tissue manipulation, the longitudinal follow up of manipulations, and in vivo imaging [49,50,51,52]. The topical application of pharmacological agents directly onto the heart or by injection into the circulation is common practice [26], as is hemodynamic manipulation through surgical procedures [29]. In fact, avian embryos are the best models for altering flow patterns at will (without introducing genetic modifications or drugs), while allowing follow up studies to study hemodynamic effects. Finally, avian embryos are inexpensive and the eggs are easy to store until ready for incubation. Because the embryos do not start developing until incubation, avian embryos allow easy planning and scheduling of experiments. Egg affordability and easy storage, moroever, facilitate experiments that require a large number of embryos. Avian models are therefore ideal to study cardiovascular development, from the onset of vasculogenesis and heart tube formation to the development of the four-chambered heart.

Limitations to avian model systems include the difficulties to perform high-throughput chemical mutagenesis screening, and the lack of stem cell technology that allows for efficient generation of genetically modified avian species. Nevertheless, the genomes of chickens and quails have been sequenced at deep coverage levels [53,54,55,56,57]. The genomic information obtained greatly helps with the design and execution of molecular perturbation experiments, including CRISPR [58,59,60], TALENs [61], RNAi [62,63,64,65], and transgenesis [36,66,67,68,69], to name a few.

## 3. Avian Development and Staging

The Hamburger and Hamilton (HH) avian staging system, originally published in 1951 and based on the chicken embryo, is the most widely used table of normal bird development [70]. It divides avian development into 46 stages: HH1 corresponds to the pre-streak embryo, prior to incubation, and HH46 is the newly hatched chick. Rather than relying on time of incubation for staging, which is imprecise and varies significantly from egg to egg, the HH staging system identifies embryos based on external characteristics, which are independent of embryo size or breed. In this way, embryos and their developmental stage can be reliably compared among studies. Early on, until about day 2 of incubation (HH1 to HH6), before somites start forming, embryonic stages are defined by the morphology of the streak and then the head formation. From HH7 to HH14, embryos are staged according to the number of somite pairs present in the embryo, as somites are clearly visible and provide a very specific and reproducible spatio-temporal pattern [71,72], with somites forming adjacent to the notochord, and sequentially from head to tail [73]. From HH14 onwards (beyond 22 somite pairs), however, counting somites becomes difficult, but limb development progresses rapidly. Embryos (HH15 to HHH29) are then staged by changes in their limbs (wings, legs) and visceral arches; and later on (HH30 to HH39) feather-germs and eyelids are also useful in staging embryos. HH40 to HH45 are based on the length of the beak and third toe, as the embryo grows but does not otherwise change significantly. In 2005, to relate embryonic development to heart development, Brad Martinsen used HH stages to describe heart developmental processes [74]. CPCs migrate to form a straight heart tube, which first manifests in the embryo by HH8+/HH9− (6–7 somites). The tubular heart starts looping by HH9+/HH10− and beating by HH10/HH11 (10–15 somites). Cardiac looping continues up to HH24. Cardiac septation occurs from HH24 to HH34 (see Table 1 for a comparison of heart developmental timings in avian species, humans and mice). At HH34, the heart has four chambers and valves, and will continue to develop until hatching.

After Hamburger and Hamilton [70] established, using chicken embryos, the prototype avian embryo staging system that occurs during sequential developmental stages, Japanese quail embryos were comparably staged according to anatomical landmarks [77,78,79,80]. More recently, the developmental stages of the quail have been further delineated using MRI technologies [81]. During the mid- to late stages of incubation, however, the accelerated rate of development in quail embryos prevents precise registration with the chicken [80].

Developmental biologists have long used the ability to biochemically distinguish between chicken and quail tissues to study developmental questions [82,83]. Quail interphase nuclei show large heterochromatic masses associated with the nucleolar RNA that stains intensely with Schiff’s reagent, whereas chicken nucleolus-associated chromatin is not significantly stained [84]. This differential staining allows quail cells to be distinguished from chicken cells in chimeric embryos. The quail-chick chimera system has been effectively used for a myriad of cardiac cell lineage analyses [83], including the seminal studies into the roles neural crest cells play in cardiovascular patterning [82,83,85,86].

## 4. Tubular Heart Assembly

**Figure 1:**
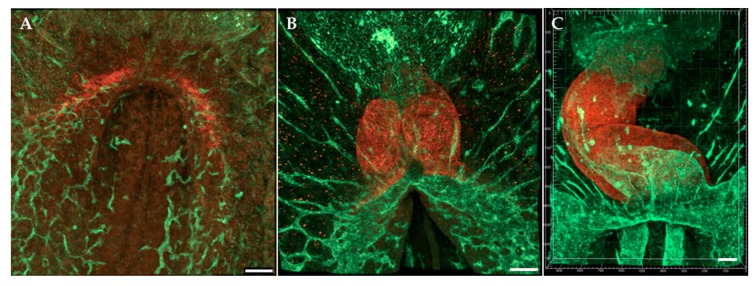
Early tubular heart formation. Antibodies (QH1 and MF20) were used to visualize endocardium and myocardium, respectively, at (**A**) HH7/HH8; (**B**) HH9; (**C**) HH12. Ventral view. QH1 (green) labels endocardial and endothelial cells; MF20 (red) labels myocardial cells. Scale bars = 100 μm.

The heart is the first functional organ in the body. The first manifestation of the heart is a linear tubular structure, termed the heart tube, that soon starts contracting. To form the heart tube, two movements of cells bring CPCs to the ventral midline, where the heart tube is formed. In the first cellular movement, heart mesoderm ingresses through the anterior primitive streak (PS) during gastrulation to form the cardiac crescent in the lateral plate mesoderm [40,87,88]. The mesoderm-derived CPCs remain in close proximity with the underlying endoderm [89,90] as they form from paired heart-forming regions of the cardiac crescent [40,91,92]. By stages HH7-HH8 (Figure 1A), the CPCs, along with their adjacent ECM, are collectively transported ventrally and converge at the midline to form the heart tube [37,38,47], which is continuous with the aorta anteriorly and the vitelline veins posteriorly. The paired heart primordia converge toward the midline, fold diagonally, and merge ventrally to the descending anterior intestinal portal (AIP), similar to the early foregut [88,93], to form the heart tube [88,89,94,95] (Figure 1B). The heart tube starts beating soon after formations (HH10) and loops (Figure 1C) and twists in preparation for later septation.

The proper fusion of the heart primordia fields and later cardiac looping requires CPC-ECM interactions [96,97,98]. At early stages, components of the primitive heart ECM include collagens I and IV, fibrillins-1 and -2, fibronectin, and laminin [98,99]. Blocking the interaction between fibronectin and integrin in avian [96,100] or mouse [101] embryos prevents the proper fusion of the cardiac primordia, leading to cardia bifida. Blocking the normal function of ECMs can also prevent proper heart field fusion or looping [102,103]. Additionally, inhibiting myosin-II-based endodermal contraction prior to HH10 impedes fusion of the bilateral heart fields toward the midline where they fuse to create the heart tube [104]. Finally, the endocardial tube forms and elongates as a consequence of differential growth, endodermal actomyosin contraction, and large-scale tissue movements that carry both CPCs and their local ECM environment into the forming heart tube [38,93,105]. Thus, ECM filaments originally associated with the anterior lateral plate mesoderm are moved medially and incorporated into the cardiac jelly [37].

The heart tube is composed of an inner endothelial (endocardial) layer and an outer myocardial layer that is separated by an ECM called cardiac jelly [106]. The myocardial layer will form the muscular walls of the heart and the muscular portions of the interventricular septum, while the endothelial layer will form the endocardium. CPCs in the cardiac crescent are unipotent and specified to give rise either to myocardial cells or to endocardial cells [107]. Endocardial precursors arise from mesodermal precursors within the cardiac crescent via the process of vasculogenesis and are subsequently arranged into a vascular tube during heart morphogenesis [107,108,109,110,111,112]. Studies in mice have suggested that the mesoderm in the cardiac crescent contains multipotent cells that can give rise to both myocardium and endocardium [113,114,115,116,117]. The endocardial-forming field, however, appears to be continuous with the vascular endothelial plexus outside of the cardiac crescent [112]. CPCs from the secondary heart field (SHF), which later contributes myocardium to the heart, appear to be either unipotent or bipotent [117]. However, it is still controversial when and where the two avian cardiac lineages and the endothelial lineage(s) are first specified and the exact location and extent of the respective precursor fields [88].

A functioning heart propels red blood cells (also known as erythrocytes) throughout the lungs and body to pick up and release O_2_ and CO_2_ at thin capillary beds. The yolk sac blood islands have long been recognized as the first site for blood cell emergence during embryonic development [118,119,120]. Undifferentiated mesoderm cells migrate from the primitive streak to the area vasculosa of the yolk sac and form aggregates called blood islands that give rise to blood and endothelial cells (ECs) in both avians [119,120] and mammals [121,122,123,124]. During primitive hematopoiesis and erythropoiesis, erythrocyte formation is tightly associated with EC formation [119], although ECs may form from small blood islands with no associated erythropoiesis [125]. Avian blood islands first appear at around HH6 in the posterior and lateral extents of extraembryonic mesoderm expansion. The earliest erythropoietic differentiation marked by hemoglobin gene expression occurs at HH7 (0–1 somite stage), which is before obvious morphological distinction between red blood cells and ECs [126]. Avian erythrocytes express a special linker histone H5 that is not seen in mammals [127,128,129]. Based on studies of continual changes in hemoglobin composition and erythrocyte morphology, primitive erythropoiesis occurs from HH6-HH27 in chicken embryos before shifting to definitive erythropoiesis [125]. The fully differentiated avian erythrocyte differs from that of the differentiated mammalian erythrocyte in that it possesses a nucleus and is capable of respiration. Despite having a nucleus, avian erythrocytes do not enter S phase or divide [130]. The mean lifespan for four species of avian erythrocytes was calculated as 39.7 ± 3.3 days (34–35 days for chickens (*Gallus gallus*) [131,132] and Japanese quail (*Coturnix japonica*) [133]). In contrast, the mean lifespan of circulating mammalian erythrocytes appears longer, calculated at 85.6 ± 10.5 days for 11 species [134] (~120 days in humans and ~40 days in mice) [135].

## 5. Cardiac Conduction

The heart begins to function while it is forming (around HH10) and pumps blood throughout the body as it continues to morph into its adult shape. The heart intrinsically generates and transmits the electrical impulse that is required to initiate synchronized cardiomyocyte contractions that propel blood throughout the body. In chick embryos, sinoatrial node cells originate in a small population of progenitor cells within the right lateral plate mesoderm posterior to the Nkx2-5+ (primary) heart field in HH5 embryos [136]. Pacemaker activity can be detected at the 7–8 somite stage (~HH9) in the sinoatrial portion (venous inflow tract (IFT) region) of the heart, even before recordings of the first heartbeat [137,138,139]. By HH9/HH10, the myocardial cells of the primitive tube are automatically and randomly conducting the electrical impulse that contracts the myocardium. This is because nascent cardiomyocytes have immature and undersized sarcomeres and sarcoplasmic reticulum, resulting in poor contraction properties. Soon, however, a contraction pattern is established and circulation begins. Initially, the rate of the heartbeat is slow and exhibits a sinusoidal electrocardiogram (ECG). The rate of the heartbeat increases as the heart develops and cardiomyocytes mature [140,141].

The primitive heart tube gives rise to the definitive left ventricle and atrioventricular canal (AVC) [47,94]. The heart tube elongates by the migration of progenitor cells from the SHF to both poles of the heart from HH14-HH18 [92,95,142,143,144,145,146,147]. The SHF-derived cells will form the definitive right ventricle, the outflow tract (OFT), the atria and the sinus venosus (SV) [143,148]. During heart tube elongation, pacemaker activity is still found in the IFT region, which suggests that cells are moving into the venous pole of the heart gain the pacemaker phenotype [149]. The firing of pacemaker cells within the sinoatrial node induces unidirectional waves of contraction that move across the expanding heart tube towards the arterial pole. Concurrently, sarcomere components and factors that regulate mitochondrial activity are upregulated, directing the cardiomyocytes in the developing chambers towards a working myocardial phenotype of fast conduction and high contractility [150]. The contractile action potential of the heart is established by HH13 in the chick [137,151]. The heart tube undergoes a torsional twist [152,153] as regions at the outer curvatures of the tube proliferate and expand to form the future atrial and ventricular chambers.

The fast conduction system of the ventricles is the final part of the pacemaking and conduction system to differentiate. In chickens, this maturation step is evidenced by the reversal in the sequence of ventricular activation [154]. The initial base-to-apex pattern of epicardial activation changes to a mature apex-to-base pattern between around HH29 to HH35. The mature “apex-first” pattern corresponds with the end of ventricular septation, and is likely to result from epicardial breakthrough near the termini of the right and left branches of the His-Purkinje system [155]. The apical activation in mice begins around E10.5, prior to the completion of ventricular septation [156]. Retroviral lineage-tracing studies in chicks indicated that main modules of the pacemaking and conduction system (e.g., the His bundle) develop independently of the other components, like the Purkinje fibers [157].

## 6. Heart Pumping and Tube Looping

**Figure 2:**
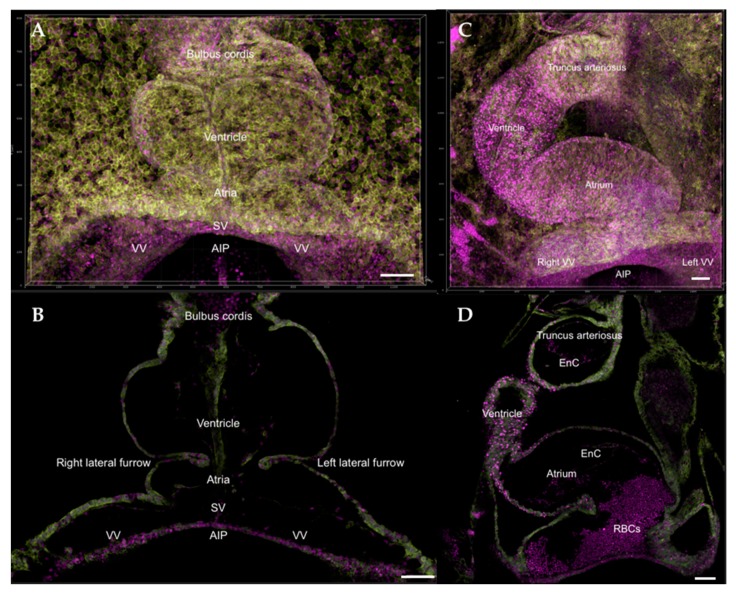
Composite 3D image of HH9 and HH14 transgenic quail heart, Tg[PGK1p.H2B-chFP; hUbCp.memb-eGFP] [67,68]. Membrane-GFP label (memb-eGFP) is shown as yellow-green, cell nuclei-cherryFP label (H2B-chFP) is shown as magenta. (**A**,**C**) Full heart at HH9 and HH14, respectively; (**B**,**D**) Single z-section within the HH9 and HH14 hearts. At HH14, red blood cells (RBCs) can be visualized from the z-section. Scale bars = 100 μm. SV, sinus venosus; VV, vitelline vein; AIP, anterior intestinal portal; RBCs, red blood cells; EnC: Endocardial cells (lining the inner tube); MCs: myocardial cells.

**Figure 3:**
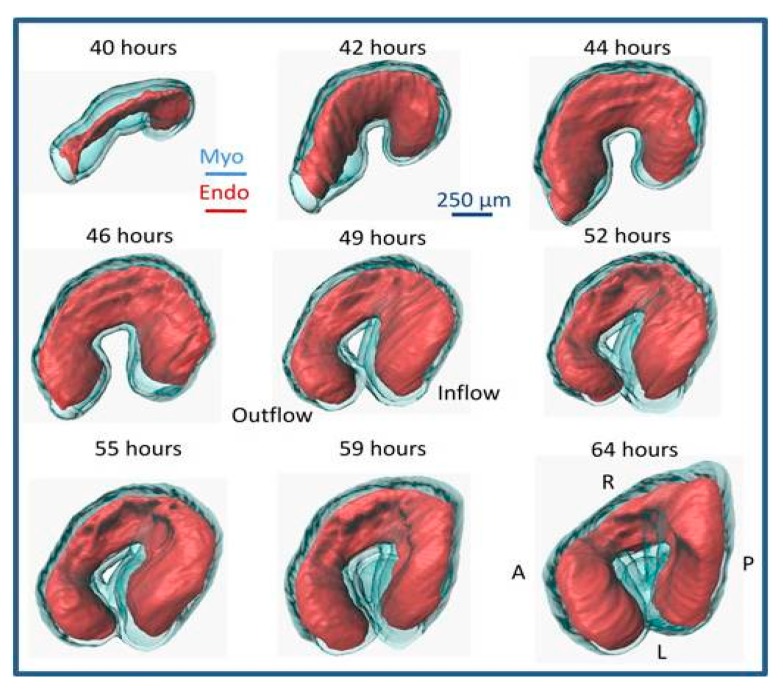
Quail heart imaged with optical coherence tomography (OCT) over looping heart stages. The pictures show normal cardiac morphological changes from 40 to 64 h of incubation (approximately HH11 to HH17), and the rightwards looping of the heart. Red (Endo): Endocardium; Blue (Myo): Myocardium. A: anterior; P: posterior; R: right; L: left. Reproduced with permission from [167].

Rightward heart looping is the first obvious sign of left–right asymmetry in avian species and mammals within the developing embryo [158] (see Figure 2, Videos S1 and S2). In day 2–3 avian embryos, and ~4 weeks human embryos, the heart tube is composed of a single anterior ventricle and a single posterior atrium. Correct heart tube looping is required for properly arranging the heart chambers and for establishing the pulmonary and systemic circulatory systems. For nearly a century, scientists have tried to understand the mechanism of heart looping by examining whether the bending component of heart c-looping is generated by asymmetric changes in myocardial cells including cell shape, cell death, cell proliferation, or space constraints (reviewed by [105,159,160,161]), but precise details are lacking. Specific molecular signaling factors from Hensen’s node during gastrulation induce the heart left–right asymmetries [162,163,164]. Recently, the lab of Angela Nieto showed that the reciprocally repressed Nodal and BMP pathways converge of their respective targets to asymmetrically activate the transcription factors Pitx2 and Prrx1, which assimilate left and right information to regulate heart laterality and morphogenesis [165]. The same group then revealed that posterior-to-anterior Nodal signals upregulate several microRNAs that transiently decrease the levels of epithelial–mesenchymal transition factors (Prrx1a and Snail1) in the left lateral plate mesoderm in a Pitx2-independent manner in the fish and mouse [166]. These findings illuminate how the Nodal and BMP pathways operate to properly balance the left-right information necessary for heart laterality and morphogenesis [166]. Also notable, the lab of Sigolѐne Meilhac used cell labelling, high-resolution episcopic microscopy, and computer simulations, to show that heart tube buckling, generated by asymmetries at the fixed arterial and venous heart poles, are sufficient to generate looping of the growing heart tube [153]. When the heart looping process ends, the initial c-shaped cardiac loop (see Figure 3) is transformed into the s-shaped loop via substantial morphogenetic events. The s-shaped heart contains the sinus venosus, the primitive atria, the primitive ventricular bend, and the primitive conus, establishing the blueprint for the formation of the multichambered heart [159]. After the looping processes finish, the region of the heart tube destined to become the atria lies anterior to the region that is destined to become the ventricles.

Endocardial cushions, which are local thickenings of the cardiac wall, form in the atrio-ventricular canal (AVC) and outflow tract of the tubular heart. Upon myocardial contraction, the cushions come into contact with each other, completely closing the lumen and limiting reverse flow [167,168,169]. Typically, endocardial cushions develop on two opposing sides of the tubular heart, such that the lumen has a shape resembling that of an ellipse [170]. Tethering proteins connecting the endocardium to the myocardium were found at regions around the lumen perimeter, where the two cushions end [167,171]. The elliptical lumen shape produced by the presence of the cushion was demonstrated to be more efficient for unidirectional pumping blood than a circular shape [172]. In fact, as endocardial cushions develop over embryonic stages, the cushions become more efficient at limiting reverse flow [173].

Cardiac cushions in the heart tube give rise to valves and septa. They are initially composed mainly of ECM, also referred to as cardiac jelly, sandwiched between the endocardium and myocardium layers. During the tubular stages of heart development, prior to valve formation and septation, cardiac cushions undergo an endocardial-mesenchymal transition (EndoMT). During EndoMT, endocardial cells delaminate from the endocardium and migrate into the cushion tissue where they proliferate and secrete ECM proteins, populating and expanding the cushions. EndoMT starts in the AVC cushions, which later give rise to the atrio-ventricular valves and septum; and later occurs in the outflow tract cushions, which gives rise to semilunar valves and a portion of the interventricular septum. The avian model has played a fundamental role in deciphering EndoMT and cardiac valve formation [174,175]. Pioneering studies by Roger Markwald and Raymond Runyan extracted AVC cushions from chicken embryos and cultured them in vitro [174,176]. Using this technique, they could study the intricate signaling pathways involved in EndoMT and thus valve formation. For example, they established that EndoMT signals originate from the myocardium [177,178,179,180]. EndoMT does not occur without the myocardium layer both in avian and mammals [181]. More recent studies, moreover, are establishing the role of blood flow on cushion EndoMT and thus valve development [182,183,184,185].

**Figure 4:**
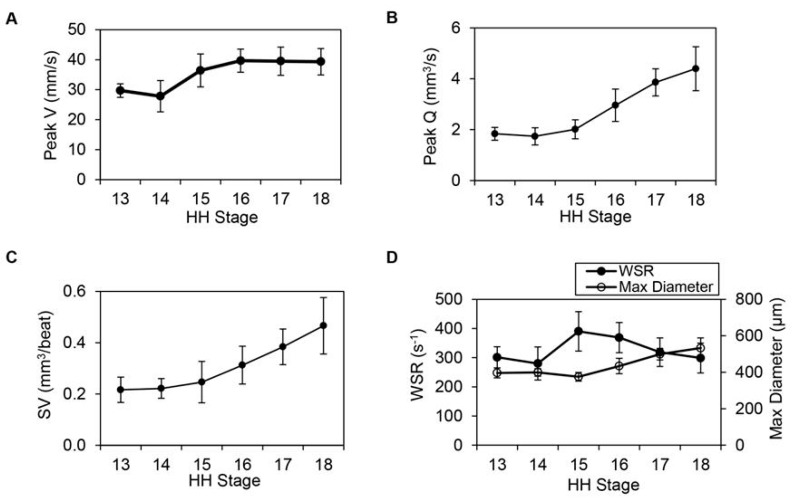
Hemodynamic data elucidating cardiac function during looping stages, from HH13 to HH18 (n = 10 at each stage). (**A**) Peak centerline velocity (mm/s), (**B**) peak volume flow rate, Q (mm^3^/s), (**C**) stroke volume, SV (mm^3^/beat) and (**D**) wall shear rate, WSR (s^−1^) with maximum diameter (mm) displayed on the secondary y-axis. Average measures are shown at each stage together with standard deviations. Reproduced with permission from [173].

Concommitant with the development of cushion, the heart becomes more efficient at pumping blood. From HH13 to HH18 (approximately 2 to 3 days of incubation), maximum centerline velocity in the heart outflow tract portion increased initially and then reached a plateau (Figure 4A). Nevertheless, volume flow rate and stroke volume increased with developmental stage (Figure 4B,C), reflecting the increasing demands of the growing embryo. An increase in volume flow rate at constant (plateaued) centerline velocity reflected an increase in outflow tract diameter (Figure 4D). Interestingly, when wall shear rate (which is proportional to wall shear stress and reflects the gradient of blood flow velocity near the wall) is approximated during the phase of maximum flow over the cardiac cycle, a characteristic trend emerges (Figure 4D). Wall shear rate increases, and this increase is followed by an increase in outflow tract diameter. Increasing diameter, however, decreases wall shear rate to previous values. This growth mechanism, in which the heart wall responds to an increased wall shear rate by increasing its diameter and thus decreasing wall shear rate to previous values, has been described for mature arteries [186,187,188], and seems to be also present at early stages of tubular heart development.

Using avian embryos, and due to their ease of access for imaging, studies have quantified early embryonic heart form and function under diverse conditions. For example, some studies quantified the changes in blood flow dynamics that occur due to early exposure to ethanol [189], trichloroethylene [190], and excess glucose [191,192]. Further, avian embryos have been used to measure changes in flow after interventions aiming at specifically disturbing normal blood flow conditions [29,193] and determined the effects of those interventions [27]. Overall, studies suggest that early heart development and the flow of blood within the heart are very susceptible to external factors during early developmental stages. Furthermore, blood flow has a pronounced effect on the development of the heart.

## 7. Heart Septation

Starting at HH25, the tubular heart enters the septation phase. During septation, the initially tubular heart will transform into a four-chamber heart with valves [74]. In the outflow tract, septation starts at the distal end, and divides the outflow tract tube into the pulmonary trunk and aorta. Pioneering work by Margaret Kirby using quail-chick chimeras demonstrated that neural crest cells, which originate from the early neural tube, migrate towards the heart and are later found in the aortico-pulmonary septum that divides the outflow tract [85]. Further, neural crest cell ablation abolishes outflow track septation [85]. The outflow tract endocardial cushions, further, will give rise to the semilunar valves. In the primitive ventricle, the interventricular septum starts growing (around HH17) progressively separating the left from the right ventricles. In the primitive atrium, an interatrial septum (the septum primum) starts to form around HH16 (E9.5 in mice) from the atrial roof. First, this septum starts as a crescent-shape ridge. By HH24, the interatrial septum has fused with the AVC cushions as they fuse to divide the AVC. In birds and mammals, in addition to the septum primum and cushions, the atrial septum is closed by tissues from the dorsal mesenchymal protrusion, which derives from the SHF [194,195]. This septation gives rise to the separation between the left and right atria, as well as the left atrio-ventricular valve (mitral valve in humans) and right atrio-ventricular valve (tricuspid valve in humans) [195]. Perforations, however, develop in the septal myocardium, which give rise to the foramen ovale, which completely closes, separating the two atria, only after hatching or birth [194]. In mammals, an additional interatrial septum is formed during development in the primitive atrium. This additional septum (septum secumdum or secondary septum) fuses with the septum primum (or primary septum) forming the mature atrial septum [195]. Thus, while avian atrial septation is slightly different than mammal atrial septation, both hearts exhibit shunting between the left and right atria, which is necessary for the fetal circulation to bypass the non-functional pulmonary system [196].

Once fully formed, the avian heart resembles the human heart, with four chambers and valves. However, the inner walls of the atria and ventricles are smoother in birds than in humans, and the avian valves are simpler than their human counterparts. The atrioventricular (AV) valves in a bird heart are different to the AV valves in humans [16]. The right AV valve consists of a single spiral flap of myocardium fastened to the inner wall of the right ventricle and thus differs from the human fibrous tricuspid valve located between the right atrium and right ventricle. The avian left AV valve, connecting the left atrium and left ventricle, is tricuspid, not bicuspid as it is in humans (mitral valve). The avian AV valves, further, are connected to the Purkinje system, a network of specialized conducting fibers composed of electrically excitable cells, which conduct the cardiac action potential that contracts the heart muscle. Like the human heart, the semilunar valves (pulmonary and aortic valves) are tricuspid. Another difference between bird and human hearts is that avian hearts are bigger (with respect to total body mass) and more muscular than mammal hearts. Regardless of these differences, avian and human heart function is remarkably similar.

## 8. The Fully Formed Heart

**Figure 5:**
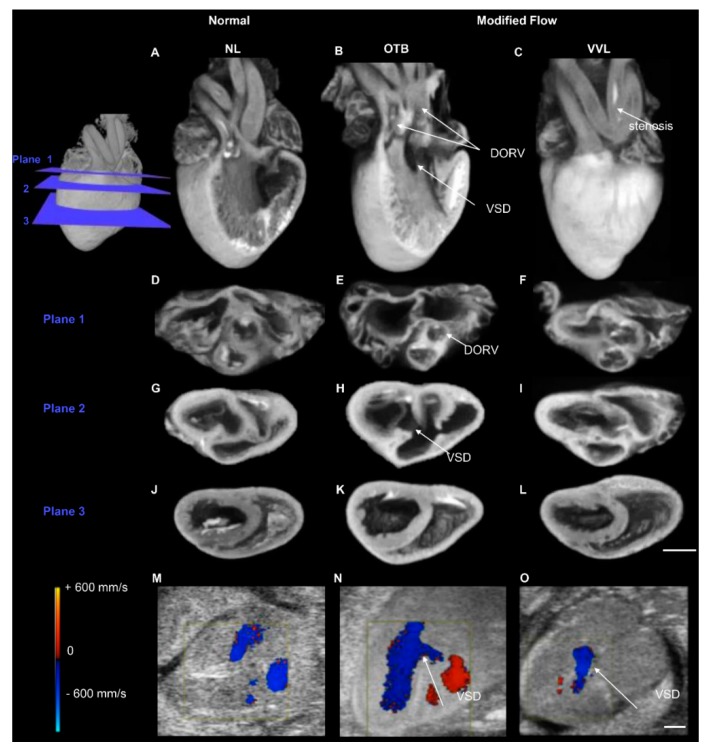
Images of the avian heart at HH38, when the heart is fully formed and can exhibit defects that are similar to those of human babies with congenital heart defects. Example microcomputed tomography (micro-CT) images of a normal heart, and two hearts with defects (generated by hemodynamic interventions – OTB and VVL). Three-dimensional reconstructions (**A**–**C**) and 3 cross-sectional planes (**D**–**L**). Plane 1 intersects the semilunar valves (**D**–**F**), plane 2 intersects the atrioventricular valves (**G**–**I**), and plane 3 intersects the ventricle midpoint (**J**–**L**). DORV in the OTB embryo displayed with aortic valve rotated outward (**E**), along with both outflows from the right ventricle and a perimembranous VSD (**B**,**H**). The VVL embryo displayed stenosis of the right brachiocephalic artery (**C**). Examples ultrasound color-Doppler images (**M**–**O**) with detection of VSD flow after both interventions (**N**,**O**). Scale bars 1 mm. NL, normal; VVL, vitelline vein ligated; OTB, outflow tract banded; DORV, double outlet right ventricle; VSD, ventricular septal defect. Reproduced with permission from [27].

One of the great advantages of using avian embryos is that early interventions can be easily followed longitudinally, and heart formation assessed. These early interventions can be, for example, transient increases or decreases in diverse bioagents, achieved by injecting or dropping compounds into the embryos, e.g., [39,189,192]. In addition, as mentioned before, cell tracing studies, using quail-chick chimeras, for example, enable researchers to determine the exact distribution of cells derived from specific progenitors upon heart formation [85,197,198]. Genetic alterations using CRISPR [58,59,60,199,200,201] or TALENs [61] are also possible. Unique to avian embryos, mechanical interventions to alter blood flow dynamics are possible, and their effect can be followed to determine how blood flow dynamics affect the formation and function of the heart [27,29,202]. Heart defects observed in human babies can be reproduced in avian embryos (see for example Figure 5). Thus, the avian embryo allows us to study heart formation in great detail—from the very beginnings of early tube formation, to the formation of the four-chambered heart.

Avian and mammalian hearts have a coronary vessel system that supplies the heart tissues with oxygenated blood. The coronary system consists of arteries and veins. The arterial coronary system originates from the ascending aorta (more specifically the sinuses of aortic valves), and then the venous coronary system returns blood to the right atrium (just inferior to the opening of the vena cava). In both avians and mammals, the coronary system runs through the surface of the heart, in the epicardium, but branches into the myocardium forming capillary networks, so that each myocardial cell is in close contact with the capillary bed [203]. The patterning of the coronary system is extremely variable in humans and not well understood. Interestingly, the epicardium and the cells that give rise to the coronary system have a different origin to myocardial and endocardial cells [203]. The epicardium has its origins in the proepicardial organ (PEO), an outgrowth of the dorsal wall of the pericardial cavity [204]. By HH18 in chicks and E10.5 in mice, the PEO contacts the surface of the developing heart in the region of the primitive atrium, and then gradually extends through the surface of the heart. In chicks, the PEO is an epithelial sheet, whereas in mice it is composed of groups of epithelial cells that eventually form a continuous sheet. The epicardium is complete by HH26 in chicks and E12.5 in mice. During epithelial migration, however, the delamination of vasculogenic precursors from the forming epicardium, an example of epithelial-to-mesenchymal transition (EMT), gives rise to the endothelial and smooth muscle cells of the entire coronary system. Interestingly, epicardial arteries form in the absence of blood flow [203]. Avian embryos, including quail-chick chimeras, played a fundamental role in elucidating the formation of the coronary system [205,206].

A fundamental difference between avian hearts and mammalian hearts is their proliferative ability after birth or hatching. In mammals, cardiomyocytes (the heart muscle cells) lose their ability to proliferate shortly after birth. During fetal stages, cardiomyocytes are mononucleated and proliferate, leading to heart growth by hyperplasia. But around the time of birth and shortly after birth, cardiomyocytes become binucleated and lose their ability to proliferate. Thereafter growth of the heart is through an increase in cardiomyocyte volume, or hypertrophy [207]. The cardiomyocytes of chickens also proliferate before hatching, with peaks of proliferation around HH20-23 [208]. However, avian cardiomyocytes continue to proliferate long after hatching [209]. Interestingly, avian cardiomyocytes start to binucleate after hatching, but even binucleated cells continue to proliferate [209]. Thus, in avian species, cardiac growth after hatching is both due to hyperplasia (proliferation) and hypertrophy (change in cell volume). The ability of avian (and reptile) heart cells to proliferate brings the potential for cardiac regeneration, although more research is required to fully understand how regeneration can be replicated in humans.

During fetal stages, when the lungs are not functional, blood is shunted from the right (pulmonary) circulation to the left (systemic) circulation. In mammals this is accomplished by the ductus arteriosus, which connects the pulmonary arteries to the aorta. In avians, in contrast, there are instead two ductus arteriosi, the left ductus arteriosus and the right ductus arteriosus [210]. Both circulations, in addition, have a right to left atrial shunt, the foramen ovale. When lung oxygenation is established right after birth or hatching, increased oxygen leads to the closure of these right–left shunts [210].

A summary of similarities and differences between avian and mammalian hearts, as presented in this review, follows (Table 2).

## 9. Imaging Strategies to Capture the Heart Beating Motion

Imaging has played a fundamental role in studies of avian (and mammal) heart development. Time-lapse imaging has been repeatedly used (with different imaging modalities) to understand heart tube formation. However, time-lapse imaging is particularly challenging in the developing and functioning heart (after HH10). Tissue growth and movements relatively slowly alter heart morphology compared to the rapid shape changes induced by the heartbeat. This is compounded by limitations in imaging systems, which are generally not fast enough to capture images of the 3D heart beating motion, also known as 4D images (3D images over time). One way to address this difficulty is to slow or stop the beating heart through cooling [211] or pharmaceutical interventions [212]. However, these approaches alter normal development and normal fluid flow in the developing heart, and can lead to information loss about the unperturbed heart shape and the dynamics of heart function. Another way is to use a signal (blood flow or ECG) to trigger image acquisition at a precise phase in the cardiac cycle, acquire 2D images over time at different locations along the heart, and then reconstruct the images in 4D. However, early during development, signals from embryonic measurements are weak and imprecise, resulting in inaccurate reconstructions of cardiac motion. To overcome these limitations, in vivo imaging and image reconstruction workflows have been developed and used to capture the dynamic three-dimensional beating motion of the developing heart using retrospective image registration algorithms [213,214,215,216,217]. These approaches acquire 2D images over time at different locations along the heart, but each 2D time series starts at a random phase in the cardiac cycle. Image registration algorithms are then used to reconstruct the 4D images. Such non-invasive heart imaging techniques allow researchers to jointly investigate heart function [173,218], morphogenesis [214,219,220,221,222], or disease [27,192] in a manner that does not require stopping or slowing the heart, nor relying on weak signals. These approaches have been employed to acquire 4D images from multiple imaging modalities such as confocal or two-photon microscopy, optical coherence tomography (OCT), and ultrasound (echocardiography), the latter in mice. Four-dimensional imaging allows for investigating the dynamics of beating, and how the dynamics is affected by development. Further, 4D imaging allows the determination of how diverse interventions (chemical, mechanical, genetic) affect heart development and the dynamics of the heart beating motion.

**Figure 6:**
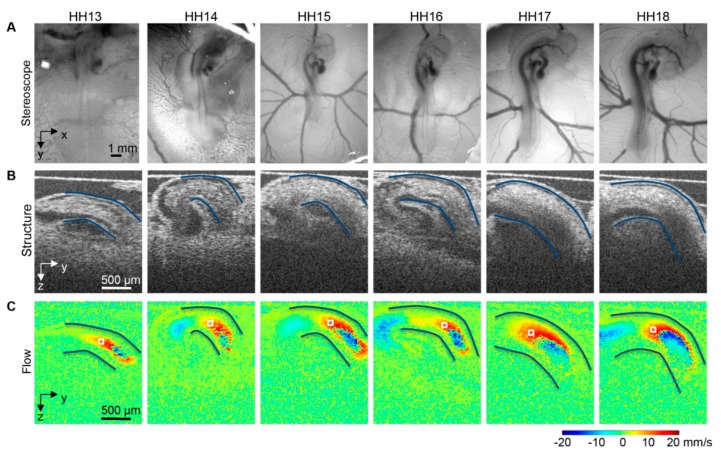
Images of the avian heart over looping stages, HH13-HH18. (**A**) Optical images of chicken embryos in ovo on the top of the egg surface, (**B**) OCT structural two-dimensional longitudinal images of the heart outflow tract and neighboring structures and (**C**) corresponding Doppler OCT images. The heart outflow tract myocardial walls are outlined in (**B**,**C**), and an example corresponding point for velocity extraction and measurement is marked by a box in (**C**). Reproduced with permission from [173].

Given the ease of access of avian embryos in ovo or ex ovo, diverse imaging modalities are employed for longitudinal studies. In avian embryos, cardiac looping can be longitudinally imaged with optical microscopy, laser microscopy (confocal, two-photon, light sheet), and OCT (see for example Figure 6). Optical microscopy is simple, in the sense that it does not require specialized equipment, and in that it allows not only the follow up of cardiac looping, but also the physical marking of different regions of the heart to determine strains and how the tissues physically bend during looping. In fact, early studies marked the heart tube and follow the marking to appreciate tube twisting [223], and to quantify heart wall strains (deformations) during cardiac expansion and contraction [224,225]. While these studies are also possible in mammals, the embryos have to be extracted for visualization, and as a result longitudinal follow up is limited, as it is limited in avian ex ovo cultures as well. In ovo non-invasive or minimally invasive imaging of avian embryos, on the other hand, allows long-term follow up as long as temperature and humidity are maintained [226,227,228].

OCT has been extensively used to study early heart development [229,230,231]. Because OCT relies on light, using the natural optical properties of tissues for contrast, it is ideal for imaging the embryos and their hearts during early embryonic stages, when the tissues are semi-transparent and the embryos are small. OCT allows for non-contact tomographic imaging with up to 2 μm resolution and up to 1–2 mm penetration without disturbing the developing embryo. Using OCT, it is possible to longitudinally follow heart looping from the beginning of tubular heart formation to the end of the looping phases [173,232,233]. However, imaging of the whole beating heart is restricted only to the first 24 h (see Figure 3), because after that OCT penetration is not enough to image the inflow portion of the heart. OCT can simultaneously acquire structural images (Figure 6B) and Doppler images (Figure 6C), which quantify the velocity component in the direction of the light beam (vertical direction in Figure 6C). Thus, OCT uniquely allows for quantifying cardiac form and function during early stages of tubular heart development [168,173,231,232,233,234].

After about HH24, the avian embryo starts sinking into the yolk and its heart size becomes too big to image with OCT. At these stages, however, echocardiography (ultrasound and Doppler ultrasound) can be employed. The resolution of research echocardiography systems, about 30 μm, is enough for imaging the embryonic heart at HH24 and beyond. It is worth mentioning that it is also possible to use echocardiography to image the hearts of mammal models, including mice, in utero, at the latest stages of development, when the embryos are big enough for echocardiography to resolve the heart structure. Ultrasound imaging allows us to visualize and measure the heart dynamics in long and short axes, enabling quantifications of stroke volume, area shortening fraction, as well as wall contraction velocities. Doppler ultrasound can be employed to quantify blood flow velocities over the cardiac cycle and compute volume flow rate, as well as determine rates of strain in cardiac walls.

To more precisely determine heart morphology, the heart can be excised and stained for histology or microCT imaging. Histological images of thin slices (spanning the whole heart) can then be reconstructed to generate the 3D morphology of the heart. Because histological sections show different aspects of tissue composition, they can be used to determine distributions of ECM components, or cell (including cell distributions and orientations within the heart). Using microCT, a 3D detailed morphological image of the heart is generated automatically, and there is no need for reconstruction. Three-dimensional microCT and histological images reveal morphological heart malformations. For example, in vivo ultrasound imaging followed by microCT imaging (see Figure 6) can be used to assess cardiac function and morphology, and can not only determine the presence of heart malformations, but also how those malformations affect heart function and even further maturation of the heart before the embryo hatches.

## 10. Summary and Conclusions

Avian embryos have been a favorite animal model for centuries, due to the simplicity by which embryos can be accessed, manipulated, and longitudinally studied inside the egg. New imaging technologies have brought new insights into the development of the embryo and its heart, allowing researchers to carefully trace the fate of progenitor cells, and the consequences of missing progenitor populations. Moreover, cell trajectories can be followed in vivo, to observe how the heart and other organs are formed and how progenitor cells continue to contribute to organ formation. In the heart, cardiac function can be monitored and evaluated over developmental stages, allowing researchers to carefully map the morphological development of the heart and the changes in blood flow conditions that accompany cardiac growth and morphological heart changes. New technologies, such as CRISPR-Cas9, and TALEN-mediated gene inactivation, are further allowing researchers to genetically modify the avian embryos and test the effect of gene disruptions, which for a long time could only be done in mice and zebrafish embryos through gene knockout or knockin lines. Transgenic quail fluorescent lines allow careful in vivo monitoring of cell fate, especially over the early developmental stages, but also the visualization of heart cell populations at later stages, including using light sheet microscopy at mature stages of heart development to determine distribution of specific cells or ECM components. While avians are not mammals, and their metabolism is higher than human metabolism, much can still be learnt from studying avian development that is very applicable to human developmental health. Basic research into how the heart develops and functions in birds provides critical insight into how the human CVS can adapt to extreme conditions be altering heart form and function. While perhaps less favored nowadays due to advances in mice genetic manipulations, avian embryos should not be left out from research endeavors. Developmental processes are highly conserved among amniotes, and avian embryos offer an uncomplicated window into human development and pregnancy inside their shelter in the egg.

## Figures and Tables

**Table 1:** Comparison of developmental timings in avian species, humans and mice.

Event	Avian Hamburger-Hamilton Stages	Human Post-Ovulatory Days	Mouse Days Post-Coitum
Formation of heart tube	HH9	~22D	E8
Heartbeat onset	HH10	~22D	E8.5
Tubular heart looping	HH10-HH24	22D–30D	E8–E10
Valve formation	HH24-HH34	37D–47D	E12–E17
Coronary system formation	HH18-HH26	33D–16 weeks	E10.5–E12.5
Atrial septation	HH16-HH34	41D–44D	E10.0–E14.5
Ventricular septation	HH19-HH34	37D–44D	E11.5–E13.5
Outflow tract septation	HH25-HH34	30D–47D	E11.5–E13.5
Fully formed heart	HH34	16 weeks	E15.5

Data based on the following references: Avian [70,74]; Human [75]; Mouse on [76].

**Table 2:** Comparison of avian and mammalian hearts.

Heart Similarities	Heart Differences
Hearts have to support resting and aerobic activities	Birds have higher metabolic needs. Resting heart rate faster in avian than humans and mice
Four-chambered heart	Chambers are larger, more muscular, and smoother in avian than mammals; heart size relative to body mass is larger in birds
Four heart valves. Semilunar valves (pulmonary and aortic valves) are tricuspid in both avian and humans	Right AV valve is a single spiral flap in birds, fibrous tricuspid valve in humans. Left AV is tricuspid in avian, but bicuspid in humans (mitral valve)
Coronary system runs through the surface of the heart, in the epicardium, and branches into the myocardium	The proepicardial organ that gives rise to the epicardium is continuous in birds, forming a sheet; but consists of groups of epithelial cells in mice that eventually form a continuous sheet
Transport of blood includes pulmonary and systemic circulations, with separation of oxygenated and deoxygenated blood	Right aortic arch develops in avian; left aortic arch in mammals
Foramen ovale closes after hatching or birth	Atrial septum formed by the septum primum and dorsal mesenchymal protrusion in birds; in mammals, in addition, there is a septum secundum that also contributes to atrial septation
Ductus arteriosi close after hatching or birth	Paired ductus arteriosus in avians (left and right ductus arteriosus); single ductus arteriosus in mammals
Cardiomyocytes, the heart muscle cells, proliferate during developmental stages in both avian and mammals increasing the heart size	Shortly after birth, mammal cardiomyocytes binucleate and stop proliferating. Further heart growth is due to volume increase. Avian cardiomyocytes continue to proliferate after hatching, and binucleate at slower rates than mammals. Binucleated avian cardiomyocytes can proliferate. Cardiac growth is due to both proliferation and volume increase
Red blood cells are present in both avian and mammals to supply oxygen to organs	Red blood cells are nucleated in avian; not nucleated in mammals, but with a larger time span than in avians

## Supplementary Materials

The following are available online at https://www.mdpi.com/2308-3425/7/1/8/s1, Videos S1 and S2: Multispectral 4D imaging of heart formation and c-looping in transgenic quail embryos.
